# Stakeholder perspectives on the governance and accountability of Nigeria’s Basic Health Care Provision Fund

**DOI:** 10.1093/heapol/czae082

**Published:** 2024-08-24

**Authors:** Mary I Adeoye, Felix A Obi, Emily R Adrion

**Affiliations:** Global Health Policy Unit, School of Social and Political Science, University of Edinburgh, 15a George Square, Edinburgh EH8 9LD, United Kingdom; Results for Development Institute (R4D), Nigeria Country Office, 31 Adamu Ciroma Crescent, Jabi District, Abuja 900108, Nigeria; Global Health Policy Unit, School of Social and Political Science, University of Edinburgh, 15a George Square, Edinburgh EH8 9LD, United Kingdom; Global Brain Health Institute, Trinity College Dublin, GBHI Office; Lloyd Building Trinity College Dublin, Dublin 2, D02 X9W9, Ireland

**Keywords:** Governance, accountability, Nigeria, health systems, primary care, UHC

## Abstract

In recent decades, Nigeria has implemented a number of health financing reforms, yet progress towards Universal Health Coverage (UHC) has remained slow. In particular, the introduction of the Basic Health Care Provision Fund (BHCPF) through the National Health Act of 2014 sought to increase coverage of basic health services in Nigeria. However, recent studies have shown that health financing schemes like the BHCPF in Nigeria are suboptimal and have frequently attributed this to weak accountability and governance of the schemes. However, little is known about the accountability and governance of health financing in Nigeria, particularly from the perspective of key actors within the system. This study explores perceptions around governance and accountability through qualitative in-depth interviews with key BHCPF actors, including high-level government officers, academics and Civil Society Organizations. Thematic analysis of the findings reveals broad views among respondents that financial processes are appropriately ring-fenced, and that financial mismanagement is not the most pressing accountability gap. Importantly, respondents report that accountability processes are unclear and weak in subnational service delivery, and cite low utilization, implicit priority setting and poor quality as issues. To accelerate UHC progress, the accountability framework must be redesigned to include greater strategic participation and leadership from subnational governments.

Key messages

- Nigeria has faced significant governance challenges within its Basic Health Care Provision Fund (BHCPF), adversely affecting the delivery of effective services and limiting access to healthcare for vulnerable populations.
- In the National Health Insurance Scheme Gateway, stakeholders felt that there is need for transparent and streamlined financial processes, and improved accountability at the sub-national levels to ensure funds reach the intended beneficiaries.
- Stakeholders viewed the recently enacted National Health Insurance Authority Act and involvement of Civil Society Organizations in BHCPF governance as positive steps towards political accountability. However, challenges remain, including limited citizen and local government involvement, impacting oversight and monitoring.
- Inclusive decision-making processes and active involvement of local communities are viewed as essential to improved accountability, as are ensuring clear mandates at each tier of government. Continued research and support are necessary to monitor effectiveness and ensure progress towards universal health coverage in Nigeria.

## Introduction

The Nigerian government has long endeavoured to attain Universal Health Coverage (UHC) for its population. A lower-middle income country of 211 million people (World Bank, 2021), its gross domestic product (GDP) of $440 billion could, in theory, provide a solid basis for progress towards UHC. However, other African nations with lower GDP have made greater progress towards UHC (Abubakar *et al*., 2022); Egypt’s UHC index, a WHO measure capturing key dimensions of UHC attainment, stands >70%, while that of Nigeria stands at 38% (WHO, 2023). Due to the underfunded healthcare system and poor health insurance coverage (Onwujekwe *et al*., 2019b; Alhassan *et al*., 2021), high out-of-pocket expenditure persists, representing ∼70% of total healthcare expenditure in the country (Aregbeshola and Khan, 2021). Other metrics suggest further room for improvement: life expectancy at birth is just 55 years, among the worst in the world (World Bank, 2020), and Nigeria accounts for nearly 20% of maternal mortality rates globally (WHO, 2019). These figures raise important questions around how the Nigerian health system is financed, managed and regulated, and highlights that greater GDP may not result in improved health outcomes.

Nigerian policymakers have implemented several reforms to address these challenges. Some examples include the Saving One Million Lives programme launched in 2012 to increase use of long-lasting contraceptives and support adequate nutrition (Oyeyemi *et al*., 2022), the Subsidy Reinvestment and Empowerment Programme (SURE-P)—Maternal and Child Health Project, also launched in 2012, which sought to improve health centres for pregnant women and neonates (World Bank, 2016; Asira, 2021), and the Primary Health Care Under One Roof programme, which focused on the integration, governance and management of primary healthcare (PHC) (Odutolu *et al*., 2016). However, concerns emerged with these reforms, including that they were highly input-focused with poor accountability structures to sustain the programmes, their expected outcomes, or citizens’ trust (Oyeyemi *et al*., 2022). As one example, the implementation of the SURE-P—Maternal and Child Health Project was criticized for being poorly designed and had important accountability challenges such as the omission of eligible participants from beneficiary lists, including the most impoverished (Oduenyi *et al*., 2019).

Nigeria is in the process of implementing another major reform. The Basic Health Care Provision Fund (BHCPF) aims to provide a basic package of healthcare services to all Nigerians and seeks to increase the effectiveness of PHC (Uzochukwu *et al*., 2018; Nelson, 2020). Within the reform, PHC is conceptualized in way that goes beyond simple delivery of primary care and is intended to take a more systemic approach to health that includes collaborative, intersectoral action at various tiers of government, moving towards a more expansive notion of PHC (Allen *et al*., 2023). It attempts to optimize one health facility in each local government area to deliver services to residents at pro-poor rates as a cost-effective, equitable and accessible means of making important progress towards UHC (Uzochukwu *et al*., 2018; Nelson, 2020). However, stakeholders have expressed concerns about BHCPF’s governance and therefore contribution to attainment of UHC, citing the gaps in responsibilities of various agencies involved in implementation, and insufficient oversight to ensure accountability and competence in fund administration (Uzochukwu *et al*., 2018).

The purpose of this study is to better understand the governance and accountability issues this new reform faces, by exploring the perceptions and experiences of key actors involved in Nigeria’s BHCPF. This is important, because peer-reviewed work on governance and accountability systems in PHC, and particularly in the BHCPF, is largely lacking. Only one peer-reviewed study was found that addresses accountability in the BHCPF. Through interviews with government representatives and implementing partners in Anambra state, Uzochukwu *et al*. (2018) highlighted governance challenges including corruption, trust and government transparency, particularly regarding budgetary control.

However, the dearth of literature in this area does not mean these issues lack attention or interest. Indeed, recent strategic plans produced by the Ministry of Health (Federal Ministry of Health (FMOH), 2010; NSHDP, 2018) address accountability and governance directly. These documents cite lack of resources and poor governance as leading causes of health system weaknesses (Federal Ministry of Health (FMOH), 2010) and outline persistent issues with poor governance, even following constitutional changes introducing a robust regulatory framework for health governance and policy for any laws relating to healthcare delivery (NSHDP, 2018). In addition, several authors have written about the poor implementation of these strategic plans themselves, and the related issues of ineffective governance and leadership (Uzochukwu *et al*., 2016; 2018; Eboreime *et al*., 2017; Aregbeshola and Khan, 2018; 2021; Aregbeshola, 2021; Asira, 2021).

Federal and state governments, legislators, international partners, non-governmental organizations (NGOs), civil society organizations (CSOs), and the media have all played an important role in implementing the BHCPF and possess first-hand knowledge of the challenges faced. Importantly, however, no study has sought to gather their experiences in implementing BHCPF. The purpose of this study is to begin to fill some of these gaps by exploring the perceptions and experiences of key stakeholders around the governance and accountability mechanisms in the BHCPF. These findings are crucial in order to provide more nuanced understandings of governance and accountability challenges within the Nigerian health system from the perspective of those directly involved in policy implementation and service delivery, and perhaps shift wider policy and public discourse around these issues, which often focus on the important, yet arguably narrower aspects of governance such as corruption and financial mismanagement (Mondlane *et al*., 2016; Onwujekwe *et al*., 2019a).

### Background: Nigeria’s BHCPF

Nigeria’s National Health Act of 2014 sought to address the persistent underfunding and substandard functioning of the health system and underscore the government’s commitment to UHC (Hafez, 2018; Uzochukwu *et al*., 2018; Abubakar *et al*., 2022). The National Health Act provided a legislative framework that led to the creation of BHCPF with the goal of promoting the provision of PHC services and guaranteeing access to a Basic Minimum Package of Health Services for all Nigerians (BHCPF Guide, 2020).

The Basic Minimum Package of Health Services is a set of ‘preventive, protective, promotive, curative, and rehabilitative health services’, including general consultations, medications, maternal health services, minor medical procedures and some major surgeries (BHCPF Guide, 2020, p. 17). The BHCPF serves as the main funding source for this, although it was only signed into the fiscal budget 5 years after it was established as an essential component of the National Health Act.

The BHCPF is divided into three ‘gateways’ (BHCPF Guide, 2020): the National Health Insurance Scheme (NHIS), using 50% of the funds to provide the Basic Minimum Package of Health Services in PHC centres; the National Primary Health Care Development Agency, using 45% to maintain PHC facilities, machinery and logistics, as well as healthcare worker capacity building; and the Federal Ministry of Health, using 5% to respond to health emergencies and epidemics (BHCPF Guide, 2020).

Our study focused on governance and accountability within the NHIS gateway specifically, as the NHIS is the primary gateway through which essential healthcare services are purchased on behalf of beneficiaries. The NHIS is a contributory social health insurance programme (Enabulele, 2020) that aims to serve vulnerable populations, including pregnant women, children 0–5 years, persons >85 years, people with disabilities and those with low income (BHCPF Guide, 2020).

### Theoretical background

Governance (also called stewardship) is conceptualized here as a government’s responsibility to ensure the development of strategic policy structures complemented by supervision and regulation, consensus building, legislation, adherence to system design and accountability (WHO, 2022a). The effectiveness of health system governance can depend upon the collaboration of several actors: (1) federal, state and local governments, (2) healthcare workers and their corporate bodies and (3) service-users and representatives who protect their interests (WHO, 2022b).

Importantly, unanimity on effective governance metrics has proven elusive in the academic and wider literature, with many distinct elements of public function cited as characteristics of effective governance (Grindle, 2011). However, accountability is one component consistently referenced as a crucial means of enhancing health system effectiveness, especially in low- and middle-income countries (LMICs) (Brinkerhoff, 2004; Baez Camargo, 2011). In fact, the concept of accountability has repeatedly been addressed either implicitly or explicitly in the health systems literature as pivotal to effective governance and stewardship (Cotlear *et al*., 2015), with some such as Baez Camargo (2011) arguing that accountability should be the main emphasis of efforts to promote governance in health systems The idea is that, as accountability is improved, the potential for corruption is reduced and responsiveness, equity and resource efficiency are better supported (Baez Camargo 2011).

To define accountability, the question ‘for what and to what purpose’ must be answered (Brinkerhoff, 2004; Baez Camargo, 2011). This study defines accountability as being predicated on connections between decision-makers and the service users impacted by their decisions (Maybin *et al*., 2011). Going beyond mere decision-making, accountability means that for health reforms to be effective, goals must be clearly stated alongside an explicit description of actors’ roles and responsibilities in accomplishing the goals of the reforms (Denis, 2014). The capacity to evaluate and verify whether these goals are reached provides oversight for the accountability relationship between key actors (Denis, 2014).

Brinkerhoff (2004) divides accountability into three categories: (1) financial accountability is the area of law and regulation pertaining to the distribution and monitoring of funds; (2) performance accountability relates to assessing, evaluating and improving service delivery in reforms; and (3) political accountability relates to the connection between the government and citizens, and refers to governance discourse, increasing public participation, equity considerations and trust.

## Materials and methods

This study uses in-depth, semistructured qualitative interviews to examine how key actors perceive accountability and governance structures in the BHCPF implementation. Governance and accountability have been identified as two areas crucial to understanding whether and to what extent the BHCPF will accelerate progress to UHC because it is implemented by multiple actors (Uzochukwu *et al*., 2018). A qualitative approach is ideal for gathering understandings and perceptions from diverse participants, and to seek detailed information on interviewees’ beliefs and decisions, professional ideology and perspective (Johnson, 2001).

Based on the key themes that emerged from a review of the relevant literature, an interview guide was developed capturing questions around governance, accountability and equity in the BHCPF (Gill *et al*., 2008; Bourgeault *et al*., 2010; Kallio *et al*., 2016). The interviewer (M.A.) did not strictly adhere to the interview guide, but rather took a flexible approach, adapting the questions to the different key actors’ dynamic contexts of meaning and perception. Thus, an open stance involving awareness of new meanings was adopted (Warren, 2002; Gill *et al*., 2008; Kallio *et al*., 2016). In the interviews, respondents were given the freedom to shape the meanings of crucial concepts and terms. The interviewer (M.A.) refrained from providing predefined definitions to avoid influencing or framing their responses.

### Recruitment process

The recruitment process was facilitated by staff from the organization Results for Development, an NGO focused on health, nutrition and education, with a history of collaboration with public actors, as well as local and international development partners, within Nigeria. The selection of interview participants was facilitated by staff from Results for Development after agreement was reached on the sampling criteria with the research team.

Since the BHCPF is a multistakeholder health system reform, we sought to recruit a variety of respondents with differing responsibilities and influence (Warren, 2002). This included key actors from the federal government (FG), NGO partners, CSOs, international donor organizations and media in order to reinforce or differentiate patterns in their responses (Warren, 2002).

This study received ethical approval from the University of Edinburgh’s School of Social and Political Science. After being informed about the purpose of the research, the reason for their selection, the process of the interview, the issues to be explored, the voluntary nature of their engagement and their anonymized and confidential participation through an information sheet, all 12 participants provided both written and oral consent. Furthermore, the respondents are identified by code numbers (1−*x*) and designated based on their organization category in order to maintain confidentiality and anonymity.

- Federal Government Worker (FGW1)
- NGO Representative (NGOR1)
- CSO Representative (CSOR1)
- International Donor Representative (IDR1)
- Media Representative (MR1)
- Academia Representative (AR1)

### Data collection and analysis

Due to travel restrictions and the COVID-19 pandemic, interviews were conducted online, using a secure audio and video conferencing platform. The interviews were conducted in English, with respondents also replying in English. Nvivo version 1.6.1 was used to facilitate thematic analysis based on verbatim interview transcripts. Because we sought to understand whether and how the data gathered through the interviews related to three prespecified dimensions of accountability (financial, performance and political accountability), a mix of deductive and inductive approaches were used. Each interview transcript was thoroughly examined to classify the main themes into thematic codes. Additionally, each transcript was carefully examined in a second attempt to assign different aspects of the transcripts to the codes or themes. Finally, each component was reassessed, and each segment was developed further using an indexing approach.

### Methodological limitations

There are a few important limitations of this study. First, online interviews may limit the extent to which the interviewer is able to build a rapport with respondents, as compared to in-person interviews. Another limitation is the potential for social desirability bias (Johnson, 2001). Of relevance here, the respondents may have assumed their expertise and work were being assessed since they are direct implementers and key decision-makers in the BHCPF and NHIS, and responded accordingly. Finally, it is crucial to acknowledge that the research team has made decisions around study design, analysis and interpretation of results, which are inevitably dependent on their unique perspectives and positionality vis-a-vis the population studied (Agee, 2009). Of particular note is the lead researcher’s positionality as a Nigerian with first-hand knowledge of the health system. However, lived experience status can be considered an advantage, and many researchers increasingly use qualitative interviews to investigate health policies or issues they are already acquainted with (Ellis and Flaherty, 1992; Denzin, 1997; Johnson, 2001). Hence, the lead researcher’s ability to identify nuances that others might miss is arguably the strength of the study.

## Results

The main themes that emerged relate to decentralization, priority-setting, service delivery and utilization and representation in decision-making bodies. The interviews revealed a distinct focus on deficient accountability systems in service delivery at the state and local government levels. Participants also discussed the anticipated role of the new ‘National Health Insurance Authority Act, 2022’, legislation that had been passed just a few weeks prior to the interviews. Further, findings are organized under headings of governance and the three aspects of accountability (financial, political and performance) identified by Brinkerhoff (2004).

### Governance in the NHIS gateway

Specific policy provisions, guidelines and oversight bodies were viewed by respondents as essential to ensuring appropriate governance within the gateway. Several respondents discussed the importance of a provision of the recent National Health Insurance Authority (NHIA) Act, (2022) which changed the ‘scheme’ into an ‘authority’, arguing that this would improve existing issues around governance in the NHIS gateway and therefore BHCPF implementation, with one key actor noting that the: *‘*National Health Insurance Authority Act *now has more autonomy in holding states responsible and ensuring that there are more vulnerable people enrolled in state-led insurance’*(AR1).

Several mentioned existing guidelines for BHCPF implementation, including an organic guideline that is reviewed regularly, as well as institutional and collaboration frameworks and accountability guidelines. One respondent noted that: ‘*there is a good governance policy framework in place, and because Nigeria struggles with not implementing policies, the government of Nigeria made policy translation oversight one of its top priorities when designing the BHCPF*’ (IDR1).

One key policy oversight mechanism emphasized by respondents is the BHCPF Ministerial Oversight Committee, referenced by 10 of 12 respondents, all of whom characterized this committee as an essential mechanism for governance and accountability. However, several respondents felt that the Ministerial Oversight Committee was not functioning as intended. One respondent explained: *‘they are not holding meetings as they should, which is supposed to be every three months, this is supposed to be sacrosanct, but this is not the reality’*(CSOR2).

Moreover, a common theme around governance and the Ministerial Oversight Committee was the crucial role of NGO and CSO partners to its functioning, with one respondent stating: *‘what I’ve also discovered is some time it is even the CSOs’ pressure that actually prompted them to convene some of these meetings, because sometimes in three months there would be no oversight meeting’*(CSOR1). Many actors felt that the Committee does not get appropriate data and information from implementing agencies due to inadequate coordination, although some were hopeful that with the new National Health Insurance Authority Act, coordination would improve. A respondent stated that: *‘One of the challenges the NHIS did have, and the* National Health Insurance Authority Act *is trying to correct that now, is that they have a lot of information, they’ve done a lot of work, and they have these data, but they’re not reporting the correct data to the* Ministerial Oversight Committee*’*(NGOR2).

Decentralization was another key theme. Several actors felt decentralization to states and local governments was the best approach to improve NHIS coverage for their most vulnerable populations through the BHCPF and accelerate the progress towards UHC. However, most NGO and CSO actors interviewed believed that insufficient support was provided at the state and local level for this to be realized. One respondent said: *‘the government began to deploy people to set up an agency, no clear guide on how to do your operations, they have no training, they have no technical capacity, they just appoint people and mandate setting up the agency for you to receive funds’*(NGOR2). Several actors felt governance within the BHCPF improved, but only at the highest level and, importantly, argued that this did not translate into improved service delivery for the most vulnerable at the state and local levels. Moreover, some argued this deficiency led to important accountability gaps in the implementation of the NHIS gateway of the BHCPF, thus hampering its effectiveness.

### Financial accountability

At the federal government level, financial accountability was generally viewed positively, largely due to ring-fencing and increasingly close monitoring of the use of funds as Nigeria transitions away from donor-funded healthcare. One respondent noted*: ‘the accountability framework put in place in the BHCPF is extremely tight at the federal government as there is a Directorate of the Office at the Ministry of Finance who are directly involved in the audit and bookkeeping of all BHCPF funds’*(IDR1). Another noted: ‘*when you are transitioning from using only donors’ money to more sustainable health financing that involves national government’s money, everyone will start asking questions; besides, the economy is in a very bad shape, so this fund, although not sufficient must be utilised effectively’*(AR1).

However, decentralization again became a key theme. Several respondents questioned the accountability measures put in place when the funds reach the states and PHC facilities, questioning whether there were sufficient mechanisms in place to ensure the funds provided translated into actual service use. One actor noted: *‘the challenge of accountability is more downstream, those ones actually using it. If this fund is for vulnerable people, are they the ones that eventually use it?’*(AR1). Another respondent argued that: ‘*the current system is purely focused on purchasing input, which are not being tied to specific outcomes. Therefore holding no one accountable within the states, which is also one of the things that is propagating a significant wastage’*(IDR1).

One respondent cited the bureaucracies surrounding funds utilization as a key cause of waste, noting the specific impact on patients: *‘First of all, money is not released timely to the states and when eventually released, there’s a delay in utilization, money can stay in the bank account, even after the disbursement for so many months without being utilized because of the bureaucratic bottlenecks within the state [*…*] The PHC will tell the vulnerable groups enrolled that the SSHIS* [State Supported Health Insurance Schemes] *has not paid their next tranche, so they say “we don’t have drugs for you free of charge, you have to pay”, because the money is irregular, it doesn’t come every three months, or the capitation will occasionally be available and become unavailable again’*(CSOR2).

Others highlighted financial failures at the state government level as a key factor. States are expected to contribute 25% towards financing the BHCPF as stipulated by law (BHCPF Guide, 2020). However, several states reportedly have failed to provide the required counterpart funding contribution. Some of the respondents believe that the states were not duly consulted before setting the 25% state-level contribution, noting that many states have low internally generated revenue and rely on federal allocations. A participant stated that*: ‘I think that the criteria given to states were very stringent. A lot of states do not have the funds, I have been wondering how we tweak the criteria for states so that more can access the BHCPF because this is really exacerbating inequity’*. (MR1).

Another recurrent theme was the lack of explicit priority-setting to guide how—and for whom—NHIS purchases the Basic Minimum Package of Health Services, with some respondents advocating for more clearly defined, and in some cases, narrower, definitions of the target population. One of the respondents opined: ‘We *have to reprioritize, instead of covering the vulnerable, why not choose the most vulnerable and poorest of the poor? (FGW1)*. Another stated: “*the BHCPF in itself has had challenges in implementation in that, right now, the number of Nigerians that it actually reaches is not enough with irregular funding. From what I know, currently the NHIS funds less than 2 million poor people but the “poor” terminology is not clear, who are the poor, what services can they or cannot get?”*(CSOR3).

### Political accountability

The National Health Insurance Authority Act, 2022, as a legal instrument, was cited by respondents as important in ensuring political accountability at the federal government level. In addition, many respondents felt that the structure of the BHCPF was a key factor as well, noting that the BHCPF’s top decision-makers included strategically important organizations for ensuring transparency and accountability, such as the media and CSOs, with the involvement of CSOs being constitutionally mandated. One representative noted that: ‘*this is the first time that the law has recognised CSOs as members of the governing council for BHCPF which implies that we now have legal backing to hold NHIS implementers accountable’*(CSOR1). However, other respondents questioned how much progress could be made given that local governments were excluded. A respondent noted: *‘the local governments are usually ignored, so how can you expect them to perform oversight and monitoring to ensure accountability when NHIS receives funding from the Ministry of Finance and distributes to State Insurance while skipping the local government and going directly to the PHC facility?’*(NGOR2). Another actor highlighted the lack of citizen involvement in decision-making saying: *‘the people receiving the services should be the ones to determine whether they have received the services that you want, at the time that they needed it and at the best quality’*(FGW1).

Importantly, when the subject of political accountability was raised in the interviews, issues around state counterpart funding emerged yet again. One respondent stated: *‘we discovered that some states are saying BHCPF [federal] money is coming in, they start getting tempted to reduce the financial allocations they have from their own mainline budget to PHC, service delivery or paying their counterpart funds’*(AR1). Another respondent argued that: *‘There can never be enough funds because the states are failing. I think that political accountability framework needs to focus more on the state level’*(NGOR2).

### Performance accountability

In relation to performance accountability, respondents touched on deficiencies in enrolment and quality assurance processes as key factors impeding service delivery. In particular, an inefficient central enrolment process was seen as creating further financial and equity issues: *‘they pool people in Wuse and register them in a PHC in Kubwa and they will have to pay so much on transportation. This is why utilization is low, access to care is low because states don’t understand insurance’*(NGOR2). For context, Wuse and Kubwa are two communities in Abuja, the Federal Capital Territory, that are ∼26 km apart. If a patient lives in Wuse but was enrolled in a PHC facility in Kubwa, they would eventually pay almost five times the cost of the NHIS capitation rate out-of-pocket on transport alone. Thus, a patient in this situation may opt to pay out-of-pocket for care in a health facility closer to where they live, where they are not a registered beneficiary.

Others highlighted an insufficient emphasis on the quality of healthcare services: *‘we always talk about access to health care, but it is not the end of it, there also have to be quality access to health care. So, somebody may have access to NHIS capitation, but there’s no medicine in it, or the HCWs are not present or not responsive, that’s not quality care’*(CSOR3). Some key actors who were interviewed argued that an accountability process that excludes quality is counterproductive and can undermine public trust in the health system, resulting in underutilization: ‘*some facilities, you go there with a headache and then maybe go back with diarrhoea because of the situation of the facility, that is poor accountability’* (MR1).

These results suggest that several factors are seen to influence effective governance and accountability in health sector reforms beyond corruption and fund mismanagement. Respondents argued that, even when funds are readily available, governance and accountability processes are critical to how and when those funds are allocated, who accesses the benefits, what services are covered and the quality of the services provided. As one of the respondents notes: *‘because the entire concept of BHCPF is equity, and equity means that you ensure that the people most in need have access to healthcare, we must ensure that our governance and accountability systems produce the desired outcome of financial protection’* (FGW1).

## Discussion

UHC centres on providing universal, timely access to high-quality healthcare services without financial hardship (WHO, 2010). While some view the concept as utopian (Cotlear *et al*., 2015), the path to UHC remains an important goal for many countries. In Nigeria, addressing current healthcare access challenges and high out-of-pocket expenditure is crucial (Cotlear *et al*., 2015). Intricately linked to the success of UHC reforms are the concepts of governance and accountability.

Importantly, governance and accountability are often viewed fairly narrowly in the context of African health systems, seen primarily as means of remedying the issue of corruption (Mondlane *et al*., 2016). However, our findings highlight that policymakers view governance and accountability as encompassing more than just remedies or preventive measures to address corruption and mismanagement, with governance seen as essential to ensuring efficiency and efficacy of health systems. In our examination of key stakeholders’ perspectives on governance and accountability mechanisms within the BHCPF NHIS gateway, a set of themes emerged around the broad categories of decentralization, financial accountability, political accountability and performance accountability.

In our interviews, the majority of governance concerns raised related to decentralization, including challenges around fund disbursement and matching, and the neglect of local governments in decision-making and administration. Decentralization is a challenge echoed in the wider literature, with Eboreime *et al*. (2017) and Cotlear *et al*. (2015) finding that the federal and state governments undertake significant responsibility for PHC because of underfunding and a lack of necessary technical expertise at the local level. As a result, PHC is administered by different tiers of government, with inadequately defined accountability mechanisms However, Eboreime *et al*. (2017) argue that the states themselves neglect budgetary transparency, lack effective service delivery monitoring and fail to prioritize healthcare.

Interestingly, the respondents described National Health Insurance Authority Act-enforced delegation of the NHIS gateway to the State Supported Health Insurance Schemes as a practical solution to support the effective implementation of the BHCPF. This highlights the important tension in this context, where decentralization has been viewed simultaneously as a problem and a solution to health systems governance. Indeed, these contradictory views are echoed in the wider literature, with Abimbola *et al*. (2015) suggesting that decentralization of PHC service delivery to local governments is an important way of bringing government closer to the people, allowing for local innovation and participation, and Eboreime *et al*. (2017) arguing that many challenges associated with decentralization are largely due to the insufficient financing and capacity within local governments.

Together, the informant interviews highlighted where the problem is thought to lie: the decentralized structure of the BHCPF coupled with relatively rigid top-down fiscal policies in which the federal government sets the minimum resource needs for each level of government while constraining their decision-making power was described as having a clear impact on service delivery. Limited state and local government capacity and financing has also inhibited successful service delivery. Increased flexibility in fund contributions, particularly in economically challenging times, and capacity-building and up-skilling management at the state and local levels could facilitate administration of the funds in a way responsive to the specific needs of an area. This is because local governments may be more responsive to local issues and be better positioned to carry out several accountability tasks, such as identifying the most at need and evaluating healthcare facility and healthcare worker performance.

Importantly, if decentralization and delegation are to be of benefit, the mandates of each tier of government must be very clear (Mosca, 2006; Baez Camargo, 2011). This will enhance performance monitoring and identification of accountability loopholes (Mosca, 2006; Baez Camargo, 2011). Our respondents argue the BHCPF governance model should depend on decisions made politically and strategically by the Ministerial Oversight Committee in close collaboration with state and local governments. The Ministerial Oversight Committee was viewed as integral to implementation, with buy-in and representation crucial for these decisions to be implemented at lower levels in the health system.

Looking in to the wider literature, authors such as Uzochukwu *et al*. (2018) recommend that the wastage can be considerably reduced at the PHC facilities by delegating to local officials serving as fund managers, who can function as local enforcers. Some authors (e.g. Ogundeji *et al*., 2019; Abubakar *et al*., 2022; Ezenwaka *et al*., 2022) recommend strategic purchasing, which can be implemented nationally with the goal of improving efficiency and wastage. Strategic purchasing uses an explicit priority-setting process to ensure the services purchased align with the health needs of the population and are chosen with careful consideration of which providers are paid for these services, as well as how, and how much. Ezenwaka *et al*. (2022) view strategic purchasing as a way to increase accountability by decreasing expenditure fragmentation and ensuring efficient implementation by allocating funding to priority people and services. Onwujekwe and Agwu (2022) issue a caution, however, noting that corruption is a significant obstacle to using strategic purchasing to reduce waste and achieve other UHC goals. Therefore, the BHCPF should consider anticorruption as a core component if choosing to promote strategic purchasing to improve accountability (Onwujekwe and Agwu 2022). Additionally, strategic purchasing requires well-defined benefits and a payment method that incentivizes quality improvement (Ogundeji *et al*., 2019), both issues that were raised by our respondents.

Authors such as Chalkidou *et al*. (2016) suggest creation of explicit accountability frameworks that address questions like: Who are the decision-makers? What are the criteria used to determine population coverage? Were the criteria adhered to in creating beneficiary lists? Are evaluation and monitoring mechanisms in place? How was equity defined? How are income thresholds set? Baatiema *et al*. (2020) highlight the importance of clear, multifaceted frameworks, incorporating both bottom-up and top-down approaches. In the context of the BHCPF, top-down accountability measures could include performance improvement strategies such as training programmes and refresher courses, or, where appropriate, results-based financing aimed at enhancing the competencies of healthcare providers. Bottom-up approaches could integrate local governments and community members into healthcare governance, alongside other relevant stakeholders to foster greater community ownership and therefore accountability.

As a solution to bureaucracies surrounding fund release and timely utilization, Uzochukwu *et al*. (2018), Baez Camargo (2011) and Brinkerhoff (2004) recommend a ‘vanguard committee’ at the local government level, that can supervise timely fund disbursement to PHCs and has the means, resources and training to perform such supervisory duties.

Finally, the role of citizen-led or citizen-participatory accountability was noted both in our interviews as well as being highlighted in most of the literature on other health reforms in Nigeria (Ogbuabor and Onwujekwe, 2018; Adenipekun, 2021: Eneh, 2022; Ezenduka *et al*., 2022). Some authors (Eneh, 2022; Ezenduka *et al*., 2022) have underscored the importance of involving the public in policymaking, including the content of benefit packages, or the delivery of services at the local level. Similarly, Kutzin (2013) has argued that people would be more empowered to demand their rights if they had a thorough knowledge of their entitlements, bridging the gap between the need for services and utilization. Thus, an accountability framework for the BHCPF involving active citizen participation may address concerns raised by respondents in this study.

### Implications of the National Health Insurance Authority Act, 2022

The National Health Insurance Authority Act was signed into law in May 2022 by then Nigerian President, Muhammadu Buhari (National Health Insurance Authority (NHIA) Act, 2022) who claimed that it would allow all Nigerians to access health insurance (Adepoju, 2022). A central purpose of the Act was to address the challenges of NHIS, many of which are highlighted in our results. Indeed, the National Health Insurance Authority Act represents a marked change in direction, repealing the NHIS Act, mandating health insurance coverage for all Nigerians and permanent residents, and enforcing the Basic Minimum Package of Healthcare Services. It also includes a ‘Vulnerable Group Fund’ that prioritizes the poor and vulnerable people (National Health Insurance Authority (NHIA) Act, 2022).

To address the governance gaps caused by decentralization, the new law obligates the federal government to coordinate with the states to increase healthcare access and provision (National Health Insurance Authority (NHIA) Act, 2022). Our respondents anticipated that the National Health Insurance Authority Act, 2022 would hold state governments more accountable, increasing equity and access to healthcare and reducing financial hardship for the poorest households. Moreover, the National Health Insurance Authority Act, 2022 enforces that the BHCPF must be used in compliance with the National Health Act, 2014. The BHCPF is also one of the major financing pathways for the Vulnerable Group Fund (National Health Insurance Authority (NHIA) Act, 2022).

At the time of our interviews, the National Health Insurance Authority Act, 2022 had only just been passed, and little was known about its potential impacts on the Nigerian healthcare system. While the stakeholders we interviewed were largely optimistic about the potential advances that would be made as a result of the Act, some concerns remain. Abubakar *et al*. (2022) noted that the law will only be effective if funds are distributed in a timely manner, with meaningful citizen participation, trust and transparency enforced in fund allocation, and collaboration with key actors in health. It will therefore be important for stakeholders and key actors to leverage this Act as a tool for accountability; more broadly, perhaps this new legislation could serve as an avenue for socializing accountability into all aspects of healthcare going forward.

### Updates on the National Health Insurance Authority Act

Since the enactment of the National Health Insurance Authority Act in May 2022, significant strides have been made to implement this transformative healthcare legislation in Nigeria. Notably, the government has held a series of engagements with stakeholders to sensitize them on the provisions of the new law and the implications for health insurance, particularly around mandatory health insurance coverage. In addition, a committee has been set up for the development of Operational Guidelines, which has now been approved by the Federal Ministry of Health.

While these advances are promising and suggest responsiveness to some of the core issues with the NHIS that our study identified, future research will be needed to provide deeper insights into the new law’s impact in the coming years. Ongoing support for research in this area will be essential to assess the Act’s effectiveness and outcomes and to continue to monitor progress towards UHC in Nigeria.

## Conclusions

This study investigated key actors’ perceptions of governance and accountability mechanisms in Nigeria’s BHCPF. The BHCPF is a UHC reform with a specific focus on financial protection for the poor and vulnerable in Nigeria. This study highlights a view among key informants that governance and accountability extend beyond simply a focus on eliminating corruption in health financing. In fact, the majority of our key informants agreed that misappropriation of funds was not a critical challenge, particularly at the national level. We therefore argue that demands for greater accountability may require a new kind of systemic accountability framework with distinct performance standards to support progress towards UHC. Such a framework must be multifaceted, incorporating both bottom-up and top-down approaches, balancing expanded local community engagement with national and state government oversight to ensure equitable and responsive health services. In the future, the challenge will be for subsequent iterations of the National Health Insurance Authority Act to include development of a governance and accountability framework that goes beyond simply ringfencing funds and includes a thorough process that reflects actionable steps for realizing progress towards UHC.

## Data Availability

The data underlying this article cannot be shared publicly to ensure the privacy of individuals who participated in the study. The data may be shared on reasonable request to the corresponding author.

## Reflexivity statement

The authors include two women and one man spanning multiple levels of seniority, all of whom work in the areas of health systems and health policy. Two of the authors, including the lead author, are Nigerian, were educated in both Nigeria and the UK and have a close familiarity with the study setting and context; one author was educated and trained in the USA and Western Europe. The lead author, M.A., is an early career researcher and young advocate for universal health coverage whose current work centres on maternal and child health, health systems strengthening and vaccine access in LMIC contexts; she previously spent >5 years working with local NGOs in Nigeria. The second author, F.O., has over 15 years of experience working on public health and universal health coverage initiatives in Africa, serving as Nigeria country manager at the organization Results for Development (R4D), where he works closely with the Nigerian national and state governments, with a specific concentration on Nigerian healthcare financing. This study was carried out as part of the lead author’s postgraduate dissertation in conjunction with R4D in Nigeria.
